# Development and validation of serum bilirubin nomogram to predict the absence of risk for severe hyperbilirubinaemia before discharge: a prospective, multicenter study

**DOI:** 10.1186/1824-7288-38-6

**Published:** 2012-02-01

**Authors:** Costantino Romagnoli, Eloisa Tiberi, Giovanni Barone, Mario De Curtis, Daniela Regoli, Piermichele Paolillo, Simonetta Picone, Stefano Anania, Maurizio Finocchi, Valentina Cardiello, Lucia Giordano, Valentina Paolucci, Enrico Zecca

**Affiliations:** 1 Department of Pediatrics, Division of Neonatology, Catholic University of the Sacred Heart, Rome, Rome; 2 Department of Pediatrics, Division of Neonatology, Sapienza University, Rome; 3 Division of Neonatology, Casilino General Hospital, Rome; 4 Neonatal Unit, San Filippo Neri, Rome; 5 Neonatal Unit, Fatebenefratelli San Peter Hospital, Rome

**Keywords:** newborn babies, hyperbilirubinemia, jaundice, predictive nomogram

## Abstract

**Background:**

Early discharge of healthy late preterm and full term newborn infants has become common practice because of the current social and economic necessities. Severe jaundice, and even kernicterus, has developed in some term infants discharged early. This study was designed to elaborate a percentile-based hour specific total serum bilirubin (TSB) nomogram and to assess its ability to predict the absence of risk for subsequent non physiologic severe hyperbilirubinaemia before discharge.

**Methods:**

A percentile-based hour-specific nomogram for TSB values was performed using TSB data of 1708 healthy full term neonates. The nomogram's predictive ability was then prospectively assessed in five different first level neonatal units, using a single TSB value determined before discharge.

**Results:**

The 75 ^th ^percentile of hour specific TSB nomogram allows to predict newborn babies without significant hyperbilirubinemia only after the first 72 hours of life. In the first 48 hours of life the observation of false negative results did not permit a safe discharge from the hospital.

**Conclusion:**

The hour-specific TSB nomogram is able to predict all neonates without risk of non physiologic hyperbilirubinemia only after 48 to 72 hours of life. The combination of TSB determination and risk factors for hyperbilirubinemia could facilitate a safe discharge from the hospital and a targeted intervention and follow-up.

## Background

Early discharge of healthy late preterm and full term newborn infants has become common practice because of the current social and economic necessities [1-3].

The association between early discharge and the need for readmission has been frequently reported, mainly because of unexpected severe hyperbilirubinemia [1,4,5].

In fact, severe jaundice and even kernicterus has developed in some term infants discharged early [6-8]. For these reasons the detection of infants without risk of severe hyperbilirubinemia has become one of the most intriguing challenges for neonatologists. However, the ability of physicians and other health care providers to recognize clinically significant jaundice and predict bilirubin levels based on the cephalocaudal progression of jaundice is limited [9-12]. Total serum bilirubin (TSB) or Transcutaneous bilirubin (TcB) determination is often the only way to avoid such difficulty but the reliability of a single TSB/TcB value to identify newborn infants at risk of significant hyperbilirubinemia is not univocally accepted [13-18]. Moreover, some authors suggested caution in applying hour-specific nomograms elaborated on different newborn population [19]. In this study, we aimed to design a percentile-based hour-specific TSB nomogram in healthy full term neonates in the first four days of life and to verify its clinical reliability in a prospective multicenter study involving five neonatal units of our region in order to identify infants without risk for subsequent severe hyperbilirubinemia and to achieve a safe neonatal discharge.

## Materials and methods

The study was conducted in 2 phases. In phase one 1708 healthy full term infants were studied to assess the normal neonatal trend of TSB with the aim to elaborate the percentile-based hour specific nomogram. In phase two, the predictive ability of the nomogram was assessed in a large study population from five different neonatal units of our region.

### Phase one - Elaboration of the predictive model

During a seven months period ended on December 2008 a total of 2147 live births took place in our institution but only 1708 healthy newborn infants were considered for the study because 439 did not meet criteria for enrolment. Exclusion criteria were prematurity, congenital anomalies, Rh or major ABO isoimmunization indexed by a positive direct antiglobulin test, or the need of intensive care. Infants presenting with delayed meconium emission (> 24 hours), hypoglycemia, hypothermia, cephalohaematoma, cutaneous bruising, hemorrhagic disease of the newborn (vitamin K deficiency), urinary tract infection, and suspected clinical sepsis were also excluded. All included babies were full term (gestational age ≥ 37 weeks), appropriate for gestational age (birth weight > 10^th ^centile), delivered by vaginal birth or caesarean section after uneventful pregnancy, without asphyxia (Apgar score ≥ 7 at 1 and 5 minutes). No drugs were administered to the infants, except for 1 mg vitamin K (Konakion, Roche Laboratories, Nutley, NJ) intramuscularly soon after birth. Feeding was started at 1 hour of life, followed by breast-feeding or bottle-feeding every 3 hours in case of low breast milk intake. No prophylactic intervention for hyperbilirubinemia was employed. Environmental lighting was constant during the study period. The infants were eligible for discharge 72 hours after birth in case of vaginal delivery and 96 hours in case of caesarean section. After obtaining parental informed consent, TSB was measured on capillary blood (OHC Photo-Ictometer, model IV, O'Hare & Co., Ltd., Tokyo, Japan) at 12 hours of life and then every 12-24 hours during the first three day of life or when clinically indicated. Newborn babies with TSB values > 15 mg/dl were discharged after a TSB decrease at two consecutive samples. In these infants direct acting bilirubin measurement was also performed.

Table 1 shows the clinical characteristics of the infants considered to perform hour-specific nomogram. Mean gestational age was 39.3 ± 1.3 weeks (range: 37-42) and mean birth weight was 3.302 ± 432 grams (range: 2580-4720); the male to female ratio was close to one; two thirds of the infants had been vaginally delivered and the majority was totally breastfed. Eighty nine neonates (5.2%) had TSB value > 15 mg/dl, while only 51 (3.0%) exceeded the value of 17 mg/dl. For newborn infants treated with phototherapy according to the American Academy of Pediatrics [20] we considered only pretreatment values. The highest TSB values showed a Gaussian distribution. Three hundred fifty (20.5%) infants reached their highest value between 24 and 48 hours of life, 837 (49.0%) had the highest TSB value between 49 and 72 hours, while 364 (21.3%) and 157 (9.2%) newborn infants reached their highest value from 73 to 96 hours and from 97 to 120 hours of life, respectively. The correlation between the TSB value obtained at single hourly determination during the first 96 hours of life and the highest absolute value was calculated using the linear regression analysis. No significant correlation between the TSB value in the first 12-24 hours of life and the highest TSB level was observed. On the contrary, there was a significant correlation between TSB values measured from 25 hours onwards and highest TSB levels (p < 0.001). Data were analyzed by using SPSS 15.0 for Windows (SPSS, Chicago, IL). TSB percentiles for each designated time were calculated, and these data were used for the design of an hour specific nomogram (Table 2) with Microsoft Excel (Microsoft, Redmond, WA).

**Table 1:** Clinical characteristics of newborn infants studied to perform hour-specific nomogram.

Variables	Neonates (1708)
Gestational age (wks)	
37	234 (13.7)
38	325 (19.0)
39	364 (21.3)
40	453 (26.5)
41	284 (16.6)
42	48 (2.8)

Birth weight (g)	
≤3000	350 (20.5)
3001-3499	697 (40.8)
3501-3999	521 (30.5)
≥ 4000	140 (8.2)

Male/Female	943/765 (55.2/44.8)
Mode of delivery	
Vaginal	1085 (63.5)
Cesarean section	542 (31.7)
Vacuum extractor	81 (4.8)

Feeding	
Exclusive breast feeding	1304 (76.3)
Breast + bottle feeding	143 (8.4)
Bottle feeding	261 (15.3)

Values expressed as number (%)

**Table 2:** Values of TSB corresponding at the 50^th^, 75^th ^and 90^th ^percentile of the hour-specific nomogram elaborated in our population.

Hrs	50^th^	75^th^	90^th^	Hrs	50^th^	75^th^	90^th^	Hrs	50^th^	75^th^	90^th^
**24**	**6.1**	**7.5**	**8.9**	49	9.0	10.3	11.9	73	10.0	11.7	13.2

25	6.2	7.7	9.1	50	9.1	10.4	12.0	74	10.0	11.8	13.3

26	6.4	7.8	9.2	51	9.1	10.4	12.1	75	10.1	11.8	13.3

27	6.5	8.0	9.3	52	9.2	10.5	12.2	76	10.1	11.8	13.4

28	6.7	8.2	9.5	53	9.2	10.6	12.3	77	10.2	11.9	13.4

29	6.8	8.3	9.6	**54**	**9.3**	**10.7**	**12.4**	**78**	**10.2**	**11.9**	**13.5**

**30**	**7.0**	**8.5**	**9.7**	55	9.3	10.8	12.5	79	10.3	12.0	13.5

31	7.2	8.6	9.9	56	9.3	10.8	12.5	80	10.3	12.1	13.6

32	7.3	8.7	10.1	57	9.3	10.9	12.6	81	10.4	12.1	13.7

33	7.5	8.9	10.2	58	9.4	10.9	12.7	82	10.5	12.2	13.7

34	7.7	9.0	10.5	59	9.4	11.0	12.8	83	10.6	12.3	13.8

35	7.9	9.1	10.6	**60**	**9.5**	**11.0**	**12.9**	**84**	**10.6**	**12.4**	**13.8**

**36**	**8.0**	**9.2**	**10.8**	61	9.5	11.1	12.9	85	10.6	12.4	13.9

37	8.1	9.3	10.8	62	9.5	11.1	12.9	86	10.7	12.5	14.0

38	8.2	9.4	10.9	63	9.5	11.2	12.9	87	10.7	12.5	14.1

39	8.3	9.5	10.9	64	9.6	11.2	13.0	88	10.7	12.5	14.2

40	8.4	9.6	11.0	65	9.6	11.3	13.0	89	10.8	12.6	14.3

41	8.5	9.7	11.1	**66**	**9.6**	**11.3**	**13.0**	**90**	**10.8**	**12.6**	**14.4**

**42**	**8.6**	**9.8**	**11.1**	67	9.6	11.4	13.0	91	10.9	12.7	14.5

43	8.7	9.9	11.2	68	9.6	11.4	13.1	92	11.0	12.9	14.6

44	8.7	9.9	11.3	69	9.7	11.5	13.1	93	11.2	13.0	14.7

45	8.8	10.0	11.5	70	9.8	11.6	13.1	94	11.3	13.2	14.8

46	8.9	10.1	11.6	71	9.8	11.7	13.2	95	11.4	13.4	14.9

47	8.9	10.2	11.7	**72**	**9.9**	**11.7**	**13.2**	**96**	**11.5**	**13.5**	**15.0**

**48**	**9.0**	**10.2**	**11.8**								

### Phase two - Prospective application of the predictive model

In phase two the predictive ability of an hourly TSB value measured between 24 to 96 hours was assessed using percentile-based hour-specific nomogram and based on the frequency of any subsequent significant severe hyperbilirubinemia defined as TSB value > 17 mg/dL, or as need for phototherapy treatment according to AAP guidelines [20].

During a 10-month period, ending in December 2009, a multicenter prospective study was conducted in five neonatal units of Rome. The study involved 2167 neonates with gestational age > 34 weeks, based on postmenstrual date confirmed by clinical assessment. Infants with congenital anomalies and those requiring neonatal intensive care were excluded from the study. All infants received 0.5 - 1 mg of vitamin K intramuscularly or orally soon after their birth. No prophylactic intervention for hyperbilirubinaemia was employed. The measurement of TSB was performed in jaundiced newborn infants and/or just before the discharge from the hospital. In all newborn babies blood samples (75 μl) for the measurements of TSB were collected by heel stick puncture. Capillary tubes were protected from light exposure and after centrifugation they were assayed with the direct spectrophotometer (Microbilimeter Dual Beam Plus model 11144A73G, Ginevri, Rome, Italy) within 30 minutes. Studied infants were never discharged before 72 hours of age independently from the mode of delivery. All newborn babies with a predischarge TSB value > 75^th ^percentile of our nomogram were discharged only after two consecutive decreasing TSB values, 12 hours apart, making us able to identify the maximum TSB level. Newborn infants with pre-discharge TcB level between the 50^th ^and the 75^th ^percentile were discharged and controlled 48 hours later for hyperbilirubinaemia. Parents of infants with TSB < 50^th ^percentile were counseled to return to the hospital within 5 days after discharge, or even earlier if persistent jaundice was observed. The decision to use phototherapy was made by the attending neonatologist according to AAP guidelines [20]. For babies exposed to phototherapy we considered only pre-treatment measurements. All perinatal data were recorded in a single database for each site with a selected log of any event occurring during the study period. Care was taken that the same clinical protocol study, method for sample collection and strategies for patient recruitment were prospectively maintained, so that the data from each unit could be pooled.

In our percentile-based hour-specific nomogram the TSB measurements were plotted on our nomogram separately by two researchers (CR and GB) after completion of the study. Statistical analysis was performed using Student's *t-*test for continuous predictors and Fisher's test for categorical data. We calculated the sensitivity, specificity, positive predictive value (PPV) and negative predictive value (NPV) plotting TSB data in the 50^th^, 75 ^th ^and 90^th ^percentile of our TSB nomogram. Special care was given to evaluate the ability of our nomogram in predicting infants without risk for subsequent severe hyperbilirubinemia. Receiver operating characteristic (ROC) curve analysis was performed with SPSS software, which was used to assess the predictive ability of our TSB nomogram.

## Results

Table 3 shows the baseline characteristics of the study population. 184 neonates (8,5%) were late preterm and there was a slight prevalence of males. The mean ± SD value for birth weight was 3237 ± 471 g (range 2000-5090) and for gestational age 38.9 ± 1.5 weeks (range 35-42). Babies were predominantly Caucasian (90.1%) and uniformly coming from caesarean section or vaginal delivery. Exclusive breast feeding was prevalent, but 39.1% of babies received also bottle feeding during their hospital stay. The mean age for TSB sampling was 63 ± 21 SD hours and the mean TSB value was 9.4 ± 0.6 mg/dL. Significant hyperbilirubinaemia, defined as TSB value > 17 mg/dL or as need for phototherapy was diagnosed in 55 newborns (2.5%): 46 neonates required phototherapy while 9 newborn babies reached a TSB value greater than 17 mg/dL but were not treated. Direct acting bilirubin was normal in all infants with TSB > 15 mg/dL. No exchange transfusion was performed and no case of significant hyperbilirubinaemia was documented after discharge.

**Table 3:** Baseline characteristics of the study population included in phase 2.

Variables	Neonates (2167)
Gestational age (wks)	38.9 ± 1.5
> 37 wks	1983 (91.5)
35 - 36 wks	184 (8.5)
Birth weight (g)	3237 ± 471
Small for gestational age	115 (5.3)
Male	1137 (52.5)

Race	
White	1952 (90.1)
Asian	106 (4.9)
Black African	55 (2.5)
Hispanic	54 (2.5)

Mode of delivery	
Vaginal	1159 (53.5)
Cesarean section	953 (44)
Vacuum extractor	55 (2.5)
Apgar score	
< 7 at 1'	53 (2.4)
< 7 at 5'	0

Feeding	
Exclusive breast feeding	1267 (58.5)
Breast + bottle feeding	848 (39.1)
Bottle feeding	52 (2.4)
Weight loss > 10%	127 (5.9)
Age at TSB (h.)	63 ± 21
TSB value (mg/dL)	9.4 ± 0.6
Significant hyperbilirubinemia	55 (2.5)
Required phototherapy	46 (2.1)
TSB > 17 mg/dL	9 (0.4)

Values expressed as mean ± SD or number (%)

Sensitivity, specificity, PPV and NPV of percentiles of our TSB nomogram in predicting significant hypebilirubinaemia are listed in table 4. Using the 75^th ^percentile 100% of sensitivity was reached only after 72 hours, while three false negative results were obtained between 24-48 hours and one false negative result has been observed between 49-72 hours. The use of the 50^th ^percentile predicted all newborn without subsequent significant hyperbilirubinaemia after 48 hours while there was only one false negative result between 24-48 hours.

**Table 4:** Ability of TSB measurements over the 50^th^, the 75^th ^and the 90^th ^percentile of TSB nomogram to predict significant hyperbilirubinaemia, for designated time periods.

Hours of age	TP (n)	FN (n)	TN (n)	FP (n)	Sensitivity (%)	Specificity (%)	PPV (%)	NPV (%)
**24 to 48 hours**								

**< 50^th ^percentile**	24	1	357	263	96.0	57.6	8.4	99.7

**< 75^th ^percentile**	22	3	479	141	88.0	77.3	13.5	99.4

**< 90^th ^percentile**	12	13	576	44	48.0	92.9	21.4	97.8

**49 to 72 hours**								

**< 50^th ^percentile**	23	0	657	368	100	64.1	5.9	100

**< 75^th ^percentile**	22	1	860	165	95.7	83.9	11.8	99.9

**< 90^th ^percentile**	17	6	975	50	73.9	95.1	25.4	99.4

**73 to 96 hours**								

**< 50^th ^percentile**	7	0	322	145	100	69.0	4.6	100

**< 75^th ^percentile**	7	0	409	58	100	87.6	10.8	100

**< 90^th ^percentile**	6	1	446	21	85.7	95.5	22.2	99.8

TP True Positive; FN False Negative; TN True negative; FP False Positive; PPV Positive Predictive Value; NPV Negative Predictive Value

**Figure 1:**
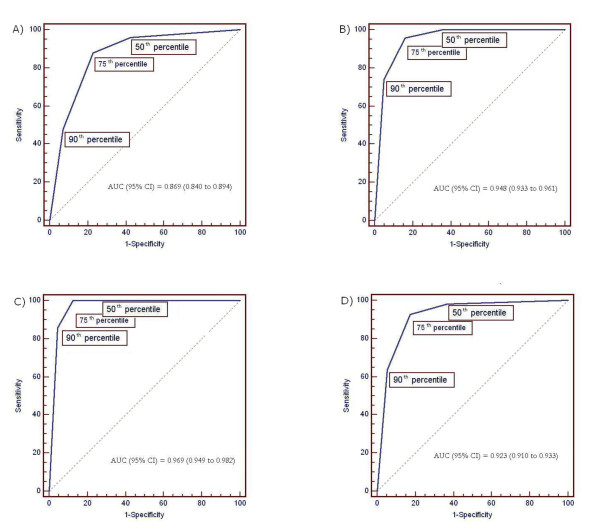
**ROC curves during different hours of age: A) 24-48 h; B) 49-72 h; C) 73-96 h; D) 24-96 h**. (AUC area under the curve).

**Figure 2:**
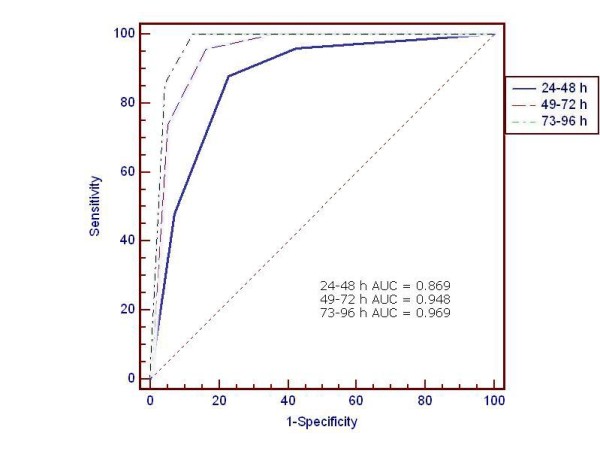
**Comparison of the ROC curves during different hours of age**.

Figure 1 depicts the ROC curves during different hours of age. The AUC measures the accuracy of the 50^th^, the 75^th ^and the 90^th ^percentile in predicting significant hyperbilirubinaemia. AUC increased gradually with hours of age (0.869 for 24-48 h; 0.948 for 49-72 h; 0.969 for 73-96 h). Figure 2 shows the comparison of the ROC curves during different hours of age.

Bhutani hour specific nomogram was applied in our study population (table 5).

**Table 5:** Ability of TSB measurements over the 40^th ^and 75^th ^percentile of Buthani nomogram to predict significant hyperbilirubinaemia, for designated time periods.

Hours of age	TP (n)	FN (n)	TN (n)	FP (n)	Sensitivity (%)	Specificity (%)	PPV (%)	NPV (%)
**24 to 48 hours**								

**< 40^th ^percentile**	25	0	238	382	100.0	38.4	6.1	100.0

**< 75^th ^percentile**	21	4	447	173	84.0	72.1	10.8	99.1

**49 to 72 hours**								

**< 40^th ^percentile**	22	1	732	293	95.7	71.4	7.0	99.9

**< 75^th ^percentile**	17	6	963	62	73.9	94.0	21.5	99.4

**73 to 96 hours**								

**< 40^th ^percentile**	7	0	374	93	100	80.1	7.0	100

**< 75^th ^percentile**	6	1	451	16	85.7	96.6	27.3	99.8

We found no difference between the predictive ability of our 50^th ^percentile and that of the 40^th ^percentile of Bhutani (table 6). The sensitivity of our 75^th ^percentile was greater than the one obtained with the 75^th ^percentile of Bhutani, and this difference was more evident between 49 and 72 hours (1 vs. 6 false negative results).

**Table 6:** Comparison between the predictive ability of our percentiles and those of Buthani.

Hours of age	TP (n)	FN (n)	TN (n)	FP (n)	Sensitivity (%)	Specificity (%)	PPV (%)	NPV (%)
**24 to 96 hours**								

**< 40^th ^percentile of Buthani nomogran**	54	1	1344	768	98.2	63.6	6.6	99.9

**< 50^th ^percentile of our nomogram**	54	1	1336	776	98.2	63.3	6.5	99.9

**< 75^th ^percentile of Buthani nomogram**	44	11	1861	251	80.0	88.1	14.9	99.4

**< 75^th ^percentile of our nomogram**	51	4	1748	364	92.7	82.8	12.3	99.8

TP True Positive; FN False Negative; TN True negative; FP False Positive; PPV Positive Predictive Value; NPV Negative Predictive Value

## Discussion

Early discharge is making the jaundice management quite difficult, since hyperbilirubinaemia is one of the main reasons for hospital readmission [1,4,5,19,20]. Recent reports suggest an increased occurrence of kernicterus in otherwise healthy newborns in North America, Western Europe and less developed part of the world [8,21-25]. In its 2004 guidelines, the AAP recommended every newborn be assessed for risk of severe hyperbilirubinaemia before hospital discharge. Two methods have been validated: the hour specific bilirubin measurements (using serum or transcutaneous determinations) and the jaundice clinical risk factor assessment. These methods can be used individually or jointly for a pre-discharge risk assessment for subsequent severe hyperbilirubinaemia. The evaluation of jaundice is now facilitated by the availability of different nomogram for both serum [26] and transcutaneous bilirubin [16,27-31]. Although there is good evidence that TcB provides excellent estimates of the TSB level, TcB should be used more as a screening tool in order to reduce the number of TSB measurements needed in the nursery than as a substitute of TSB [32,33]. Bhutani and colleagues demonstrated a strong relationship between the hour specific TSB level and the risk of subsequent hyperbilirubinaemia [26], but concerns regarding the use of pre-discharge TSB values were expressed as a consequence of false negative results [17]. Such false negative results have been described but their exact frequency is unknown. Two studies of infants readmitted for hyperbilirubinaemia showed that 2.7% to 3.6% of babies had a pre-discharge bilirubin level in the low-risk zone (< 40^th ^percentile) while 13.5% to 43% of babies were in the low-intermediate risk zone (40-75^th ^percentile) so showing a significant risk of false negative results [18,34]. The rate of false negative and the different performances of Bhutani nomogram as a predictive tool can be explained by racial, genetic and environmental factors that affected the course of neonatal hyperbilirubinaemia. A nomogram cannot perform well in every setting if the background between the published and the tested population is too much different. Moreover, predictive tools should be developed in one sample and validated in another one [35]. The use of Bhutani nomogram as a screening tool has been questioned because of concerns regarding its reliability on different population and some methodological flaw of the study from which it was developed (i.e. follow-up bilirubin levels not performed in more than 75% of study participants, and the lack of validation in an independent patient sample)[17]. Bhutani's nomogram was generated in a retrospective study including infants from a single urban Pennsylvania hospital, so the demographic, racial, genetic and environmental features of that population may not adequately represent other newborn populations.

To reach the aim of our study we previously developed our hour-specific percentile based nomogram using serial measurements of TSB in a cohort of healthy full term neonates. In the second phase we verified prospectively its predictive ability to identify newborn at risk for significant hyperbilirubinaemia (defined as TSB > 17 mg/dL or need for phototherapy) in a multicenter study involving a large neonatal population. The

sensitivity of the 50^th ^percentile was 100% between 49 and 96 hours, while it was affected by one false negative result before 48 hours of age. Using the 75^th ^percentile as risk discriminator we obtained 88% of sensitivity between 24 and 48 hours (3 false negative results) and 95.7% of sensitivity between 49 and 72 hours (one false negative result). The false negative between 49 and 72 hours was a preterm infant who had his TSB measurement at 70 hours of age and subsequently needed phototherapy because he reached a TSB value of 16 mg/dL. This observation suggests that a risk assessment strategy combining the pre-discharge bilirubin risk zone and some clinical factor (such as gestational age) could have a better overall predictive accuracy than a strategy using pre-discharge bilirubin risk zone by itself [36-39].

In a subsequent analysis we plotted TSB measurements of our study population on Bhutani hour specific nomogram and we found that the sensitivity and the specificity of our 50^th ^percentile were the same as the 40^th ^percentile of Bhutani. Instead our 75^th ^percentile performed better than the 75^th ^percentile of Bhutani (sensitivity 92.7% vs. 80%) especially between 49 and 72 hours of age (sensitivity 95.7% vs. 73.9%).

The strengths of our study are the large sample size, well representing the demographic characteristics of Italian neonatal population, the high follow-up rate, and its robust design. It is also noteworthy that it is a prospective observational study in which enrolled babies were studied during their hospital stay, thus avoiding sampling bias. All newborns referred to their birth hospital for clinical follow up after discharge, and no readmissions because of hyperbilirubinaemia were noticed. Our study has some limitations: firstly, the incidence of significant hyperbilirubinaemia is low; secondly it is not a population based study but the newborns were enrolled according to the clinical practice of each neonatal unit.

## Conclusion

Our percentile based TSB nomogram is a useful tool for predicting infants without subsequent severe hyperbilirubinaemia before discharge. There is a notable difference in the false positive rate with the Bhutani's nomogram, especially between 49 and 72 hours of age. It has to be emphasized that the use of pre-discharge bilirubin screening alone may falsely reassure physicians about a safe early discharge of the mother-neonate pair. Bilirubin level should be assessed together with clinical risk factors (such as gestational age, exclusive breastfeeding, east asian race, cephalhematoma, significant bruising or previous sibling with jaundice) to determine infant risk for subsequent severe hyperbilirubinaemia, thus ensuring an appropriate follow up.

## Competing interests

The authors declare that they have no competing interests.

## Authors' contributions

CR and EZ designed the study and has made substantial contributions in drafting manuscript. ET, MDC, SP, SA, MF and VC performed data acquisition and validation for every single participating center. GB, DR and LG contributed to interpretation of data and performed statistical analysis. PP and VP verified data analysis and revised the manuscript critically. All authors read and approved the final manuscript
